# Impact of Kidney Function on Effects of the Dietary Approaches to Stop Hypertension (Dash) Diet

**DOI:** 10.4172/2167-1095.1000168

**Published:** 2014-08-05

**Authors:** Crystal C Tyson, Maragatha Kuchibhatla, Uptal D Patel, Patrick H Pun, Alex Chang, Chinazo Nwankwo, Michael A Joseph, Laura P Svetkey

**Affiliations:** 1 Department of Medicine, Division of Nephrology, Duke University Medical Center, Durham, NC, USA; 2 Sarah W. Stedman Nutrition and Metabolism Center, Duke University Medical Center, Durham, NC, USA; 3 Duke Hypertension Center, Duke University Medical Center, Durham, NC, USA; 4 Department of Biostatistics & Bioinformatics, Duke University Medical Center, Durham, NC, USA; 5 Division of Nephrology, Johns Hopkins University, Baltimore, USA; 6 Welch Center for Prevention, Epidemiology, and Clinical Research, Johns Hopkins University, Baltimore, USA; 7 Department of Epidemiology and Biostatistics, SUNY Downstate Medical Center, New York, USA

**Keywords:** Kidney disease, Diet, Blood pressure, Hypertension

## Abstract

**Objectives:**

Although the Dietary Approaches to Stop Hypertension (DASH) diet lowers blood pressure in adults with hypertension, how kidney function impacts this effect is not known. We evaluated whether Estimated Glomerular Filtration Rate (eGFR) modifies the effect of the DASH diet on blood pressure, markers of mineral metabolism, and markers of kidney function.

**Methods:**

Secondary analysis of the DASH-Sodium trial, a multicenter, randomized, controlled human feeding study that evaluated the blood pressure lowering effect of the DASH diet at three levels of sodium intake. Data from 92 participants with pre-hypertension or stage 1 hypertension during the 3450 mg /day sodium diet assignment contributed to this analysis. Stored frozen plasma and urine specimens were used to measure kidney related laboratory outcomes.

**Results:**

Effects of the DASH diet on blood pressure, phosphorus, intact parathyroid hormone, creatinine, and albuminuria were not modified by baseline eGFR (mean 84.5 ± 18.0 ml/min/1.73 m^2^, range 44.1 to 138.6 ml/min/1.73 m^2^) or the presence of chronic kidney disease (N=13%).

**Conclusions:**

The impact of the DASH diet on blood pressure, markers of mineral metabolism, and markers of kidney function does not appear to be modified by eGFR in this small subset of DASH-Sodium trial participants with relatively preserved kidney function. Whether greater reduction in eGFR modifies the effects of DASH on kidney related measures is yet to be determined. A larger study in individuals with more advanced kidney disease is needed to establish the efficacy and safety of the DASH diet in this patient population.

## Introduction

Although often underemphasized in clinical practice, a mainstay of therapy for all individuals with hypertension is lifestyle modification [1]. One such modification that effectively lowers BP in adults is the Dietary Approaches to Stop Hypertension (DASH) diet [2,3]. Benefits of the DASH diet have been established in various patient populations; however, its efficacy in individuals with reduced kidney function is not well studied. In fact, the presence of kidney disease was a criterion for participant exclusion from major DASH studies [4,5]. Because 69% of US adults with Chronic Kidney Disease (CKD) are estimated to have uncontrolled hypertension, identifying and employing multiple strategies to lower blood pressure may help improve hypertension control rates [6]. Assessing whether kidney function modifies both the BP lowering effect and safety profile of the DASH diet is an appropriate first step in determining whether this intervention is a viable treatment option for patients with comorbid CKD and hypertension.

The DASH diet is rich in fruits, vegetables, and low-fat dairy products, and low in fat, sweets, and added sugars. It emphasizes healthy sources of protein, such as, lean meats, nuts, seeds, and legumes. The resultant nutritional profile is high in potassium, calcium, phosphorus, and protein. Because high intake of these nutrients may cause significant metabolic derangements and increased morbidity in some individuals with reduced kidney function, current kidney disease outcome quality initiative guidelines do not recommend routine adoption of the DASH diet by patients with moderate disease (estimated glomerular filtration rate [eGFR] <60 ml/min/1.73 m^2^) [7]. However, mounting data suggests that a diet high in fruits and vegetables can be safely consumed by patients with early and even advanced CKD [8,9]. It is also more widely recognized that the dietary sources of phosphorus (inorganic vs. organic) and protein (animal vs. plant derived) influence the bioavailability and potential health effects of these nutrients, with organic phosphorus and plant derived protein being less harmful [10-13]. Hence, it seems appropriate to reassess the validity of traditional dietary restrictions that are often recommended for patients with CKD. The above findings support the relevancy of taking a closer look at the DASH diet, a diet high in fruits and vegetables, organic phosphorus, and plant derived protein, to determine whether it may be appropriate at reduced levels of kidney function for hypertension management.

In this secondary analysis of the DASH-Sodium trial, which includes participants with preserved or mildly reduced kidney function, we evaluated whether the BP lowering effect of the DASH diet is modified by eGFR. We also evaluated whether the DASH diet negatively impacted kidney-related laboratory measures as eGFR declined.

## Methods

The DASH-Sodium trial was a multi-center, randomized, controlled human feeding study that evaluated the effects of the DASH diet at three levels of sodium intake on BP in adults with pre-hypertension or untreated stage I hypertension. Detailed descriptions of trial methods and main results have been previously published [3,4]. An Institutional Review Board at each participating study site approved the research protocol. All participants provided written informed consent and a Data and Safety Monitoring Board provided trial oversight.

### Study population

DASH-Sodium participants were ≥22 years old with mean Systolic BP (SBP) of 120-159 mmHg and mean Diastolic BP (DBP) of 80-95 mmHg. Exclusion criteria were use of antihypertensive medications or nutritional supplements, history of cardiovascular disease, type 1 or poorly controlled type 2 diabetes mellitus, poorly controlled hyperlipidemia, Body Mass Index (BMI) >40 kg/m^2^, consumption of >14 alcoholic beverages per week, pregnancy or breast-feeding, and kidney disease. Kidney disease was defined as either a serum creatinine >1.2 mg/dl for women and >1.5 mg/dl for men (unless eGFR was ≥60 ml/min per Cockcroft-Gault equation) [14] or ≥2+ protein on urine dipstick regardless of serum creatinine.

### Trial design

After a 2 week run-in period, participants were randomized to receive either the DASH diet or a control diet in parallel group design. The composition of each diet is shown in Table 1. Assigned diets were administered for three consecutive 30-day feeding periods each at a low (1150 mg/d), intermediate (2300 mg/d), and high (3450 mg/d) sodium level in random crossover fashion. Participants were provided all of their food, including cooked meals and snacks. Energy intake was adjusted to keep weight constant during the study.

### Measurements

Our analyses included only those participants for whom both stored pre-intervention plasma and urine specimens were available, which allowed us to measure necessary labs to assess baseline eGFR, albuminuria and CKD status. We used DASH-Sodium trial data to evaluate change in BP from the beginning to the end of the 30-day high sodium (3450 mg/d) feeding period, which was the sodium intake typical of most Americans at the time and equivalent to the sodium level provided in the original DASH trial [2]. Weight at screening visit 3 was used as baseline weight. BMI was calculated by dividing weight in kilograms by height in meters squared.

### Blood pressure

Detailed description of the BP measurement protocol has been previously published [3]. BP measurements obtained during the screening and run-in phases were averaged to determine baseline BP. Those obtained during the first week of the high sodium intervention period were averaged to determine pre-intervention BP used in our analysis. Measurements obtained during five of the last nine days of the high sodium intervention period were averaged to determine post-intervention BP.

### Laboratory data

Fasting plasma specimens and 24-hour urine collections were obtained during screening and the last week of each intervention period and subsequently frozen at −70°C. We retrieved and thawed available frozen plasma specimens to measure phosphorus (colorimetric method), intact parathyroid hormone (iPTH; electrochemiluminescence immunoassay), and creatinine (kinetic Jaffe method). Urine specimens were thawed to measure albumin (immunoturbidimetric method) and creatinine concentrations (LabCorp, Burlington, NC). Frozen specimens had no prior freeze-thaw cycles. We were unable to measure plasma potassium and calcium concentrations because blood was collected into vacutainers containing Ethylenediaminetetraacetic Acid (EDTA), an anticoagulant that contains potassium salts and chelates calcium, resulting in inaccurate potassium and calcium measurements.

Estimated GFR was calculated using the Chronic Kidney Disease Epidemiology Collaboration equation [15]. Participants were classified as having albuminuria if baseline urine albumin-to-creatinine ratio (UACR) was ≥17 mg/g in men or ≥25 mg/g in women [16]. Based on a single plasma and urine sample collected during the screening period, participants were classified as having CKD if eGFR was <60 ml/min/1.73 m^2^ or if albuminuria was present at any eGFR. Participants with CKD were classified into the following standard stages: Stage 1=eGFR ≥ 90 ml/min/1.73 m^2^ with albuminuria, Stage 2=eGFR of 60-89 ml/min/1.73 m^2^ with albuminuria, and Stage 3=eGFR of 30-59 ml/min/1.73 m^2^.

### Outcomes

Primary outcomes were change in SBP and DBP during the 30 day high sodium intervention period, comparing DASH to control by level of kidney function. Secondary outcomes were changes in phosphorus and iPTH (measures of mineral metabolism) and changes in creatinine, eGFR, and UACR (measures kidney function) from the screening period to the end of the high sodium intervention period.

### Statistical analysis

Standard descriptive statistics were used to determine baseline demographics, physical measures, and laboratory data. Means, standard deviations, and ranges were computed for continuous variables and frequency counts with percentages were computed for categorical variables. Between group differences in continuous variables were compared using student’s t-test and categorical variables using chi-square test. Difference in between group changes in outcome variables was compared using general linear models. The relationship between eGFR and change in outcome variables were determined using multiple linear regressions. All models were adjusted for site, intervention period, baseline eGFR, and each respective baseline variable. Additionally, SBP and DBP were adjusted for race, gender, age and BMI. Regression models included an assessment for interaction between diet and baseline eGFR. Interactions between diet and the following variables were also assessed: evenly distributed eGFR quartiles (Q1: ≥98, Q2: 81.7-98, Q3: 72-81.7, Q4: <72), CKD stages (1-3), and CKD status (yes, no). Goodness-of-fit of the models were assessed. These were exploratory analysis and no adjustment is made for multiple testing. A significance level of 0.05 was used for all tests. Statistical analysis was performed using SAS 9.3 (SAS Institute, Inc., Cary, NC).

## Results

### Participant characteristics

There were a total of 412 participants enrolled in the DASH-Sodium trial. After exclusion of participants with missing baseline plasma or baseline urine samples, 92 (22%) individuals were included in our analysis. Compared to those who were excluded, our subset of participants were younger (46.0 ± 10.2 vs. 48.8 ± 9.7 years; p=0.02) and had a higher mean BMI (30.4 ± 5.5 vs. 28.8 ± 4.6 kg/m^2^; p<0.01). All other demographic and baseline physical measurements were similar. Table 2 shows demographic and baseline physical and laboratory measurements for participants included in our analysis, overall and by diet assignment. A majority were black (65%) and female (63%). Forty-four (48%) participants received the control diet and 48 (52%) participants received the DASH diet. Mean eGFR was 84.5 ± 18.0 ml/min/1.73 m^2^ with a wide range from 44.1 to 138.6 ml/min/1.73 m^2^ and 70% had a baseline eGFR >75 ml/min/1.73 m^2^. A total of 12 (13%) participants had CKD (eGFR <60 ml/min/1.73 m^2^ or presence of albuminuria irrespective of eGFR). Compared to those with normal kidney function, participants with CKD had a higher mean baseline plasma creatinine concentration (1.3 ± 0.3 vs 1.0 ± 0.2 mg/dl; p <0.001), lower eGFR (64.8 ± 20.2 vs 87.4 ± 15.7 ml/min/1.73 m^2^; p<0.001), greater albuminuria (128.1 ± 250.8 vs. 2.8 ± 4.1 mg/g; p<0.001), and higher mean SBP (138.8 ± 11.2 vs. 132.8 ± 9.3 mmHg; p=0.05) at study entry. Participants with CKD also tended to be older than those with normal kidney function but this was not statistically significant (50.3 ± 10.7 vs 45.4 ± 10.0 years; p=0.12).

### Blood pressure

**Figure 1:**
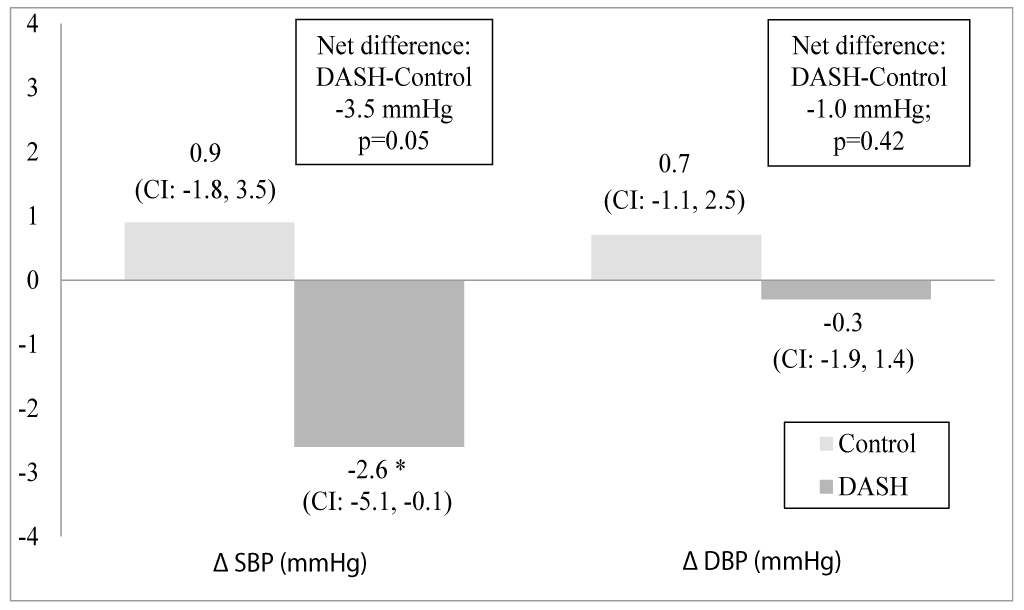
Change in blood pressure for 89 participants of the DASH-Sodium trial by diet assignment after a 4-week feeding intervention. General linear models were adjusted for site, intervention period, baseline blood pressure, estimated glomerular filtration rate, race, gender, age and body mass index. Between group difference (DASH minus Control) and p-values are offset in boxes. ^*^p=0.02. Sample size is 42 for control and 47 for DASH (minus 3 participants with missing pre- or post-intervention BP). SBP, systolic blood pressure; DBP, diastolic blood pressure; CI, 95% confidence interval; diff, difference.

After a 4-week feeding intervention, there was a significant reduction in SBP for the DASH group (−2.6 ± 9.7 mmHg; 95% CI:−5.1, −0.1 mmHg) but not the control group (0.9 ± 9.6 mmHg; 95% CI: −1.8, 3.5) in both the unadjusted (data not shown) and adjusted models (Figure 1). Between group difference for change in SBP was significant (−3.5 ± 1.7 (SE) mmHg, p=0.05). DBP did not change for either the DASH or control group in unadjusted and adjusted models. Baseline eGFR was not a significant covariate for change in SBP or DBP.

### Mineral metabolism

**Figure 2:**
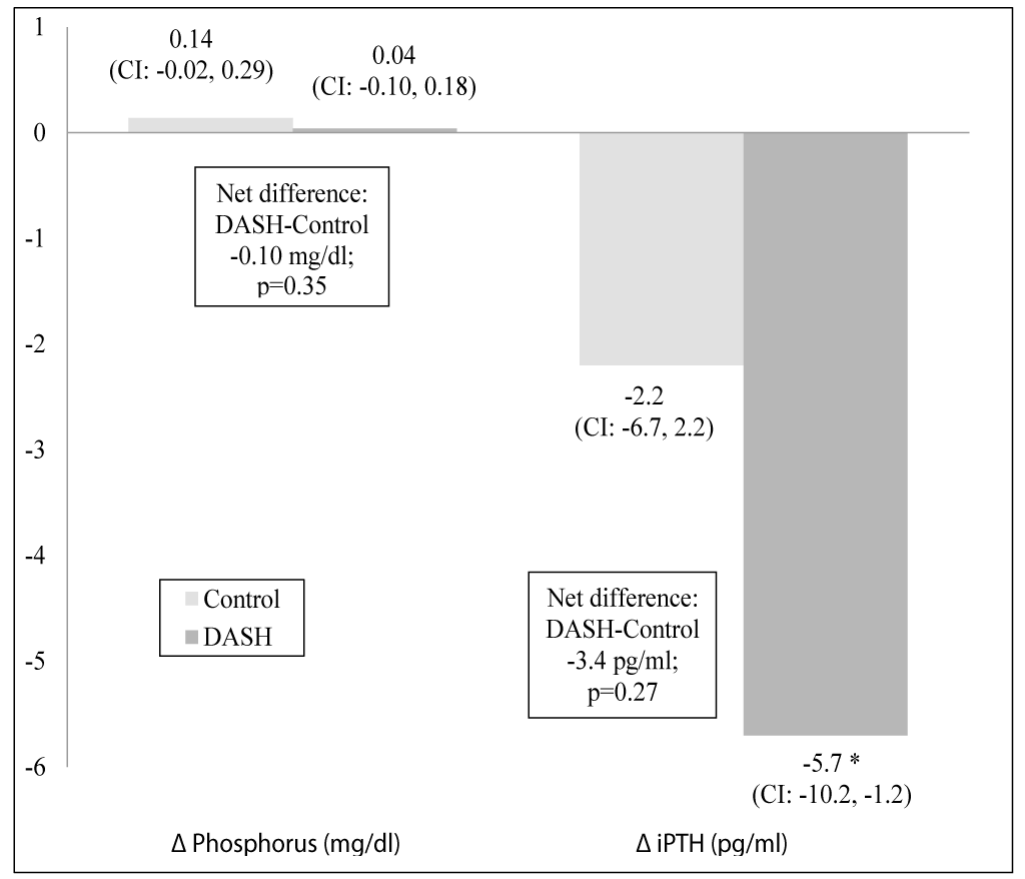
Change in measures of mineral metabolism for participants of the DASH-Sodium trial by diet assignment after a 4-week feeding intervention. General linear models were adjusted for site, intervention period, baseline estimated glomerular filtration rate, and each respective baseline value (phosphorus, iPTH). Between group difference (DASH minus Control) and p-values are offset in boxes. ^*^statistically significant. Sample size for phosphorus was 83 (38 control and 45 DASH minus 9 participants with missing pre- or post-intervention data) and iPTH was 80 (39 control and 41 DASH minus 12 participants with missing pre- or post-intervention data). iPTH, intact parathyroid hormone; CI, 95% confidence interval; diff, difference.

Plasma phosphorus did not change after the 4-week feeding intervention within or between the two diet groups (Figure 2). In the unadjusted model, there was no significant change in mean plasma iPTH for either diet group. However, after adjusting for site, intervention period, and baseline iPTH, there was a significant reduction in mean plasma iPTH in the DASH group (−5.7 pg/ml; 95% CI: −10.2, −1.2 pg/ml) but not the control group (−2.2 pg/ml; 95% CI: −6.7, 2.2 pg/ml). Between group difference for change in iPTH did not reach statistical significance (−3.4 pg/ml, p=0.27). Baseline eGFR was not a significant covariate for change in plasma phosphorus or iPTH.

### Kidney function

**Figure 3:**
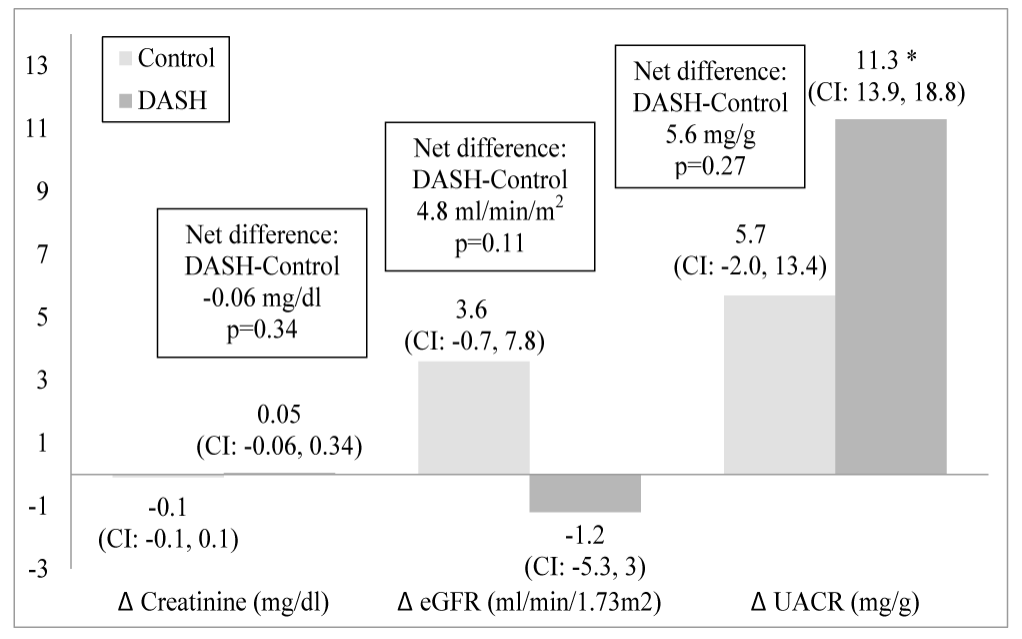
Change in kidney function for 92 participants of the DASH-Sodium trial by diet assignment after a 4-week feeding intervention. General linear models were adjusted for site, intervention period, baseline eGFR, and each respective baseline value (creatinine, eGFR, UACR). Between group difference (DASH minus Control) and p-values for participants with both preand post-intervention plasma or urine samples are offset in boxes. Sample sizes for creatinine, eGFR and UACR were 81 (control 38, DASH 43), 87 (42 control, 45 DASH), and 72 (34 control, 38 DASH), respectively. eGFR, estimated glomerular filtration rate; UACR, urine albumin-to-creatinine ratio; CI, 95% confidence interval; diff, difference.

Plasma creatinine and eGFR did not change significantly in either the DASH or control groups after the 4-week feeding intervention in both unadjusted and adjusted models (Figure 3). Between groups differences for change in these variables were also not significant. There were also no significant changes in UACR for either the DASH or control groups in the unadjusted models. However, UACR increased in the DASH group (11.3 mg/g; 95% CI: 3.9, 18.8 mg/g), but not the control group (5.7 mg/g; CI: −2.0, 13.4 mg/g) after adjusting for site, intervention period, and baseline UACR. Between group difference for change in UACR was not significant (5.6 mg/g, p=0.27). Baseline eGFR was not a significant covariate for change in creatinine or UACR; however, it was significantly related with post intervention eGFR after adjustment (beta estimate −0.30, SE 0.15, p=0.045).

### Impact of eGFR on the relationship between diet and change in outcome variables

Adjusted multiple linear regression models were performed to determine if the relationship between diet and change in each outcome variable was altered by kidney function. Separate models were performed to evaluate kidney function by baseline eGFR, eGFR quartiles (Q1-Q4), CKD stage (1-3) and CKD status (yes or no). For each of these models, the effect of the DASH diet on change in SBP, DBP, phosphorus, iPTH, creatinine, eGFR, or UACR did not vary by level of kidney function. Table 3 shows results for linear regression models for each outcome variable with coefficient estimates for eGFR, DASH diet, and interaction between eGFR and the DASH diet. There were no significant interactions between eGFR and the DASH diet (p-values >0.05).

## Discussion

In this secondary analysis of the DASH-Sodium trial, we were able to evaluate the impact of the DASH diet on BP, markers of mineral metabolism, and kidney function as well as how these relationships may be influenced by level of kidney function. After a 4-week feeding intervention in adults with pre-hypertension and untreated stage 1 hypertension, we confirmed previous findings from the DASH-Sodium trial and other feeding studies that the DASH diet lowers SBP, even in a subpopulation with mildly decreased kidney function [2,3,17]. Although we did not observe an association between the DASH diet and change in DBP, phosphorus, creatinine, or eGFR, we did observe an associated reduction in iPTH and an associated increase in UACR with the DASH diet. Effects of the DASH diet on BP, markers of mineral metabolism, and kidney function were not modified by baseline kidney function. Presence of CKD was also not observed to affect the impact of DASH on BP or kidney-related outcomes. Thus, in this small subset of DASH-Sodium participants, the DASH diet was effective in lowering SBP without major adverse effects on kidney parameters, across a wide range of eGFR.

Our aim was to determine if kidney function modified the effect of the DASH diet on BP. Current knowledge about the management of hypertension in patients with CKD, as well as the effects of the DASH diet in various patient subgroups, could lead to two opposing hypotheses. On one hand, the DASH diet may be hypothesized to have a smaller BP lowering effect as eGFR declines because patients with CKD tend to have more difficult to control hypertension than those with preserved kidney function. For example, patients with CKD have a higher prevalence of uncontrolled hypertension and require a greater number of medications to achieve BP goals when compared to those without CKD [6,18,19]. On the other hand, the DASH diet may be hypothesized to have a greater BP lowering effect as eGFR declines because previous studies have shown that it reduces BP to a greater degree when hypertension is more severe (i.e., there was greater reduction in BP for participants with hypertension than those with prehypertension) [20]. However, in the current study, neither eGFR nor the presence of CKD modified the BP lowering effect of this dietary intervention, at least for the range of eGFR assessed. This suggests that the DASH diet may have a similar effect on BP for those with mild to moderate CKD as it does for those with preserved kidney function.

Evidence that the DASH diet reduces BP is insufficient alone to recommend it to patients with CKD because evidence of its safety is also required. Unfortunately, we were unable to directly measure serum concentrations of key electrolytes, such as potassium and calcium. Our small sample size of participants with CKD further limits or ability to compare those with reduced and preserved kidney function. Presumably at a certain eGFR, the DASH diet could in fact cause a rise in these serum electrolytes; the “safe range” of eGFR is yet to be determined.

To our knowledge, an association between the DASH diet and changes in plasma iPTH or phosphorus has not been demonstrated. Under normal conditions, serum calcium and phosphorus are tightly regulated by interplay between both vitamin D and parathyroid hormones which act on bone, the intestines, and the kidneys to maintain physiologic balance. A previous study demonstrated urinary excretion of both calcium and phosphorus to be higher for participants on the DASH diet compared to the control diet, reflecting higher dietary intake [21]. Unexpectedly, we noted a significant reduction in plasma iPTH for the DASH group. We suspect that higher concentrations of serum calcium and/or vitamin D (neither of which were evaluated in this study) may explain this finding.

Estimates of renal function were not altered by the DASH diet, despite its higher protein content. Restricting protein for individuals with reduced kidney function remains controversial since important clinical trials that evaluated whether protein restriction slows disease progression were inconclusive or showed only modest benefit [22,23]. Our data suggest that a slightly higher protein intake (18% in DASH vs 15% in control) does not lead to acute reductions in kidney function in the setting of mild CKD. We did observe a modest increase in estimated daily urine albumin excretion for participants on the DASH diet compared to those on the control diet (net increase of 5.6 mg). This is likely a reflection of increased protein consumption rather than glomerular disease. However, the potential long term implications of this change will need to be established. We speculate that the BP lowering effect of the DASH diet would outweigh the potential risks of such a modest increase in urine albumin excretion.

We acknowledge that our study has some limitations. Inclusion of a small subset (22%) of participants who were enrolled in the DASH-Sodium trial potentially introduces bias from unbalanced comparison groups. Compared to participants who were excluded, our subset was younger and had a higher BMI. However, in a subgroup analysis of the DASH-Sodium trial the effect of the DASH diet did not vary by age (>45 years) or BMI (≥30 kg/m^2^) suggesting that this difference may not necessarily influence the expected impact of DASH on BP in our study [24]. Another study limitation is our inability to directly measure key electrolytes, such as, potassium and calcium, due to plasma samples containing EDTA. Assessing the impact of the DASH diet on these electrolytes in patients with CKD is essential to determine whether this intervention is a safe treatment option for hypertensive patients with CKD. Perhaps most critical, our study is limited by the range of eGFR included and classifying participants as having CKD based on a single plasma and urine sample. Only 9% had eGFR less than 60 ml/min/1.73 m^2^, none less than 44 ml/min/1.73 m^2^, and only 13% were classified as having CKD. A larger study in patients with lower kidney function is clearly needed to definitively establish the efficacy and safety of the DASH diet in this population, albeit with caution due to risk of hyperkalemia.

## Conclusion

In this small subset of DASH-Sodium participants, our findings suggest that kidney function, as measured by eGFR, does not modify the effect of the DASH diet on blood pressure or markers of mineral metabolism and kidney function. If confirmed in a larger population of patients with less preserved kidney function, the DASH diet may be a valuable, nonpharmacologic strategy for BP control in individuals with CKD.

## Figures and Tables

**Table 1:** Daily nutrient content of the DASH and control diets in the DASH-Sodium Trial.

Nutrients*	DASH	Control
Energy (kcal)	2100	2100
Total fat (% energy)	26	36
Saturated fatty acids	5	15
Monounsaturated fatty acids	13	13
Polyunsaturated fatty acids	8	8
Protein (% energy)	18	15
Carbohydrate (% energy)	56	49
Cholesterol (mg)	150	300
Fiber (g)	32	11
Potassium (mg)	4700	1700
Magnesium (mg)	500	160
Phosphorus (mg)	1700	1100
Calcium (mg)	1250	450
Sodium (mg)†		
Low sodium	1150	1150
Intermediate sodium	2300	2300
High sodium	3450	3450

* Based on 2100 kcal daily intake.

† Diets were administered for three consecutive 30-day feeding periods each at 3 levels of sodium in random crossover fashion. Only data for the high sodium (3450 mg) intervention period was used in our analysis

**Table 2:** Demographics and baseline measures for 92 participants of the DASH-Sodium trial overall and by diet assignment.

	Total (N=92)	Control (N=44)	DASH (N=48)	P value
Age, yr (SD)	46.0 (10.2)	45.8 (11.1)	46.2 (9.4)	0.86
Men, N (%)	34 (37.0)	15 (34.1)	19 (39.6)	0.59
Black, N (%)	60 (65.2)	31 (70.5)	29 (60.4)	0.31
**Household income**				
≤60,000/year, N (%)	67 (74.4)	35 (81.4)	32 (68.1)	0.15
Education				
Some college or less, N (%)	49 (53.9)	23 (53.5)	26 (54.2)	0.97
**Alcohol, N (%)**				
≤7 drinks/week	91 (99.0)	44 (100)	47 (97.9)	0.09
SBP mmHg, mean (SD)	133.6 (9.7)	134.5 (10.4)	132.8 (9.1)	0.4
DBP mmHg, mean (SD)	84.6 (4.6)	85.0 (3.5)	84.3 (5.5)	0.48
Hypertension* (yes)	34 (37.0)	15 (34.1)	19 (39.6)	0.59
Weight, baseline, kg, mean (SD)	87.6 (16.8)	89.9 (18.2)	85.5 (15.2)	0.22
BMI, kg/m^2^, mean (SD)	30.4 (5.5)	31.2 (6.0)	29.7 (5.0)	0.2
Phosphorus, mg/dl, mean (SD)	3.2 (0.5)	3.1 (0.5)	3.2 (0.5)	0.46
Intact PTH, pg/ml, mean (SD)	45.7 (17.8)	47.2 (16.9)	44.2 (18.7)	0.43
Creatinine, mg/dl, mean (SD)	1.0 (0.2)	1.0 (0.2)	1.04 (0.2)	0.41
eGFR ml/min/1.73m^2^, mean (SD)	84.5 (18.0)	86.8 (18.9)	82.4 (16.9)	0.24
**eGFR range:**				0.9
>90, N (%)	31 (34)	15 (34)	16 (33)	
75-89, N (%)	33 (36)	17 (39)	16 (33)	
60-74, N (%)	20 (22)	9 (20)	11(23)	
<60, N (%)	8 (9)	3 (9)	5 (10)	
Albuminuria† (yes), N (%)	7 (8)	5 (11)	2 (4)	0.19
CKD‡ (yes), N (%)	12 (13.0)	6 (13.6)	6 (12.5)	0.87
**CKD Stage:**				0.1
Stage 1, eGFR ≥ 90, N (%)	1 (1.1)	0 (0)	1 (2.1)	
Stage 2, eGFR 60-89, N (%)	3 (3.3)	3 (6.8)	0 (0)	
Stage 3, eGFR <60, N (%)	8 (8.7)	3 (6.8)	5 (10.4)	

* Hypertension defined as SBP ≥140 mmHg or DBP ≥90 mmHg.

† Albuminuria defined as urine albumin-to-creatinine ratio ≥17 mg/g for men and ≥25 mg/g for women.

‡ CKD defined as eGFR less than 60 ml/min/1.73 m^2^ or presence of albuminuria at any eGFR.

SBP, systolic blood pressure; DBP, diastolic blood pressure; PTH, parathyroid hormone; CKD, chronic kidney disease; BMI, body mass index; eGFR, estimated glomerular filtration rate

**Table 3:** Results for linear regression models showed no significant interaction between estimated glomerular filtration rate and the DASH diet.

	Beta Estimate (SE)	95% CI for Beta
Δ **SBP (mmHg)**		
eGFR	−0.01 (0.07)	−0.15, 0.13
DASH	−2.07 (8.63)	−19.22, 15.08
eGFR×DASH	0.02 (0.10)	−0.18, 0.22
Δ **DBP (mmHg)**		
eGFR	0.03 (0.05)	−0.07, 0.13
DASH	−3.81 (5.76)	−15.26, 7.64
eGFR×DASH	0.03 (0.07)	−0.11, 0.17
Δ **phosphorus (mg/dl)**		
eGFR	0.005 (0.005)	−0.005, 0.015
DASH	0.57 (0.52)	−0.46, 1.60
eGFR × DASH	0.01 (0.01)	−0.01, 0.03
Δ **iPTH (pg/ml)**		
eGFR	−0.13 (0.15)	−0.43, 0.17
DASH	12.66 (16.06)	−19.31, 44.63
eGFR×DASH	−0.19 (0.19)	−0.57, 0.19
Δ creatinine (mg/dl)		
eGFR	0.004 (0.010)	−0.016, 0.024
DASH	0.22 (0.43)	−0.64, 1.08
eGFR×DASH	0.002 (0.005)	−0.008, 0.012
Δ **eGFR (ml/min/1.73 m^2^)**		
eGFR	−0.30 (0.15)	−0.60, 0.00
DASH	2.44 (15.83)	−29.03, 33.91
eGFR×DASH	0.08 (0.18)	−0.28, 0.44
Δ **UACR (mg/g)**		
eGFR	0.35 (0.25)	−0.15, 0.85
DASH	−6.50 (28.69)	−63.72, 50.72
eGFR×DASH	0.14 (0.33)	−0.52, 0.80

All models were adjusted for site, intervention period, baseline eGFR, and each respective baseline measure. SBP and DBP were additionally adjusted for race, gender, age and body mass index. SBP, systolic blood pressure; DBP, diastolic blood pressure; PTH, parathyroid hormone; CKD, chronic kidney disease; eGFR, estimated glomerular filtration rate; UACR, urine albumin-to-creatinine ratio

## References

[1] Chobanian AV, Bakris GL, Black HR, Cushman WC, Green LA (2003). The Seventh Report of the Joint National Committee on Prevention, Detection, Evaluation, and Treatment of High Blood Pressure: the JNC 7 report. JAMA.

[2] Appel LJ, Moore TJ, Obarzanek E, Vollmer WM, Svetkey LP (1997). A clinical trial of the effects of dietary patterns on blood pressure. DASH Collaborative Research Group. N Engl J Med.

[3] Sacks FM, Svetkey LP, Vollmer WM, Appel LJ, Bray GA (2001). Effects on blood pressure of reduced dietary sodium and the Dietary Approaches to Stop Hypertension (DASH) diet. DASH-Sodium Collaborative Research Group. N Engl J Med.

[4] Svetkey LP, Sacks FM, Obarzanek E, Vollmer WM, Appel LJ (1999). The DASH Diet, Sodium Intake and Blood Pressure Trial (DASH-sodium): rationale and design. DASH-Sodium Collaborative Research Group. J Am Diet Assoc.

[5] Sacks FM, Obarzanek E, Windhauser MM, Svetkey LP, Vollmer WM (1995). Rationale and design of the Dietary Approaches to Stop Hypertension trial (DASH). A multicenter controlled-feeding study of dietary patterns to lower blood pressure. Ann Epidemiol.

[6] Plantinga LC, Miller ER, Stevens LA, Saran R, Messer K (2009). Blood pressure control among persons without and with chronic kidney disease: US trends and risk factors 1999-2006. Hypertension.

[7] Kidney Disease Outcomes Quality Initiative (K/DOQI) (2004). K/DOQI clinical practice guidelines on hypertension and antihypertensive agents in chronic kidney disease. Am J Kidney Dis.

[8] Goraya N, Simoni J, Jo C, Wesson DE (2012). Dietary acid reduction with fruits and vegetables or bicarbonate attenuates kidney injury in patients with a moderately reduced glomerular filtration rate due to hypertensive nephropathy. Kidney Int.

[9] Goraya N, Simoni J, Jo CH, Wesson DE (2013). A comparison of treating metabolic acidosis in CKD stage 4 hypertensive kidney disease with fruits and vegetables or sodium bicarbonate. Clin J Am Soc Nephrol.

[10] Sullivan C, Sayre SS, Leon JB, Machekano R, Love TE (2009). Effect of food additives on hyperphosphatemia among patients with end-stage renal disease: a randomized controlled trial. JAMA.

[11] Scialla JJ, Appel LJ, Wolf M, Yang W, Zhang XM (2012). Plant Protein Intake is Associated With Fibroblast Growth Factor 23 and Serum Bicarbonate Levels in Patients With Chronic Kidney Disease: The Chronic Renal Insufficiency Cohort Study. J Ren Nutr.

[12] Schlemmer U, Frølich W, Prieto RM, Grases F (2009). Phytate in foods and significance for humans: food sources, intake, processing, bioavailability, protective role and analysis. Mol Nutr Food Res.

[13] Moe SM, Zidehsarai MP, Chambers MA, Jackman LA, Radcliffe JS (2011). Vegetarian compared with meat dietary protein source and phosphorus homeostasis in chronic kidney disease. Clin J Am Soc Nephrol.

[14] Cockcroft DW, Gault MH (1976). Prediction of creatinine clearance from serum creatinine. Nephron.

[15] Levey AS, Stevens LA, Schmid CH, Zhang YL, Castro AF (2009). A new equation to estimate glomerular filtration rate. Ann Intern Med.

[16] Mattix HJ, Hsu CY, Shaykevich S, Curhan G (2002). Use of the albumin/creatinine ratio to detect microalbuminuria: implications of sex and race. J Am Soc Nephrol.

[17] Appel LJ, Sacks FM, Carey VJ, Obarzanek E, Swain JF (2005). Effects of protein, monounsaturated fat, and carbohydrate intake on blood pressure and serum lipids: results of the OmniHeart randomized trial. JAMA.

[18] Cushman WC, Ford CE, Cutler JA, Margolis KL, Davis BR (2002). Success and predictors of blood pressure control in diverse North American settings: the antihypertensive and lipid-lowering treatment to prevent heart attack trial (ALLHAT). J Clin Hypertens (Greenwich).

[19] Bakris GL, Williams M, Dworkin L, Elliott WJ, Epstein M (2000). preserving renal function in adults with hypertension and diabetes: a consensus approach. National Kidney Foundation Hypertension and Diabetes Executive Committees Working Group. Am J Kidney Dis.

[20] Svetkey LP, Simons-Morton D, Vollmer WM, Appel LJ, Conlin PR (1999). Effects of dietary patterns on blood pressure: subgroup analysis of the Dietary Approaches to Stop Hypertension (DASH) randomized clinical trial. Arch Intern Med.

[21] Lin PH, Ginty F, Appel LJ, Aickin M, Bohannon A (2003). The DASH diet and sodium reduction improve markers of bone turnover and calcium metabolism in adults. J Nutr.

[22] Klahr S, Levey AS, Beck GJ, Caggiula AW, Hunsicker L (1994). The effects of dietary protein restriction and blood-pressure control on the progression of chronic renal disease. Modification of Diet in Renal Disease Study Group. N Engl J Med.

[23] Kasiske BL, Lakatua JD, Ma JZ, Louis TA (1998). A meta-analysis of the effects of dietary protein restriction on the rate of decline in renal function. Am J Kidney Dis.

[24] Vollmer WM, Sacks FM, Ard J, Appel LJ, Bray GA (2001). Effects of diet and sodium intake on blood pressure: subgroup analysis of the DASH-sodium trial. Ann Intern Med.

